# Epidural lidocaine, butorphanol, and butorphanol – lidocaine combination in dromedary camels

**DOI:** 10.1186/s12917-023-03601-8

**Published:** 2023-02-16

**Authors:** Ayman El Nahas, Adel Ibrahim Almubarak, Usama Hagag

**Affiliations:** 1 Department of Clinical Sciences, College of Veterinary Medicine, King Faisal University, PO Box 400, Al-Ahasa 31982, Al Hofuf, Saudi Arabia; 2 Department of Surgery, Anesthesiology and Radiology, Faculty of Veterinary Medicine, Beni-Suef University, Beni-Suef, 62511 Egypt

**Keywords:** Epidural, Lidocaine, Butorphanol, Camel, Anti-nociception, Sedation, Opioid, Local anesthetic

## Abstract

**Background:**

The use of general anesthesia in dromedary camels is constrained by risks related to decubitus. Caudal epidural analgesia is an alternative convenient technique providing loco-regional analgesia for numerous invasive and noninvasive painful conditions. Lidocaine is probably the most commonly used local anesthetic in clinical practice, but has a relatively short duration and may not provide significant long term analgesic benefits. Epidural administration of an opioid-local anesthetic mixture would improve the quality and length of analgesia and minimizes the adverse motor effects provoked by local anesthetics. Butorphanol (potent agonist–antagonist opioid) has been used to improve the duration of epidural analgesia in some animal species, but not in camels. Therefore, our purpose was to investigate the onset and duration of analgesia as well as the clinical and hemato-biochemical effects produced by the epidural administration of butorphanol (0.04 mg/kg), lidocaine (0.22 mg/ kg), and butorphanol-lidocaine (0.04 mg/kg—0.22 mg/ kg) mixture in nine adult dromedary camels in a crossover experimental study.

**Results:**

The onset of analgesia was not statistically different between lidocaine (6.5 ± 2.3 min) and butorphanol-lidocaine (7.3 ± 1.5 min) combination. Delayed onset of analgesia was reported after butorphanol administration (14.7 ± 3.5 min). Butorphanol-lidocaine combination produced marked longer duration (175 ± 8.7 min) than lidocaine (55 ± 6.8 min) and butorphanol (158 ± 5.3 min). Mild ataxia was observed in the butorphanol–lidocaine and lidocaine treated animals and slight sedation was reported after butorphanol and butorphanol-lidocaine administration. A transient significant increase in the glucose levels was recorded after all treatments.

**Conclusions:**

Epidural administration of butorphanol augments the analgesic effects and duration of lidocaine with minimal adverse effects.

**Supplementary Information:**

The online version contains supplementary material available at 10.1186/s12917-023-03601-8.

## Background

Anesthesia and analgesia are mandatory when certain diagnostic or surgical procedures are implemented. The purpose is to facilitate the intended procedure and to guarantee both animal and personnel safety; however, economic considerations may dictate the use of certain technique. In ruminants, inhalation anesthesia is seldom feasible and economically justified, except for animals of high economic value. Injectable anesthesia is cost effective, easily performed and relatively safe [1]. However, ruminants including camels are not ideal patients for general anesthesia due to the possibility of tympany or passage of ruminal contents and/or saliva into the airways [2]. Epidural anesthesia is an appropriate substitute usually executed in conscious animals, avoiding the hazards of decubitus and risks of general anesthesia [3].

Caudal epidural anesthesia and analgesia is a widely employed method in ruminants with various painful invasive or noninvasive procedures [4, 5], as it is safe and provides efficient analgesia in a multitude of clinical applications [6]. It furnishes desensitization of the perineum and urogenital organs, thus it is particularly useful for performing various surgical procedures or other invasive manipulations in the conscious standing animal without pain or discomfort [7, 8]. Lidocaine is most frequently used local anesthetic for epidural anesthesia. It acts by diffusion through the nerve cell membrane, enter sodium channels, and inhibit the influx of sodium ions, thereby interrupting nerve conduction [9]. However, it offers a relatively short-term analgesic benefits and in acidic environment (purulent inflammation), the onset time and efficacy of lidocaine is either delayed or ineffective [10, 11]. In addition, lidocaine indiscriminately inhibits the sympathetic motor and sensory fibers, which can lead to motor instabilities or even recumbency [2]. Epidural administration of opioids is a more suitable option for procedures requiring long-lasting analgesia. Opioids selectively inhibit the sensory fibers resulting into considerable analgesia and lower risk of rear limb dysfunction [12].

Opioids are the main pillars of perioperative analgesia in many animal species [13] and are frequently used as adjuncts to local anesthetics in epidural anesthesia to promote the quality of blockade and lengthen the period of analgesia [14]. Butorphanol is a mixed agonist–antagonist opioid with minimal side effect profile among opioids [15] and is occasionally used in cattle, sheep, goats and camels to provide analgesia [16, 17]. Butorphanol produces its analgesic effects by interacting with κ-receptor as well as μ- and δ-opioid receptors [18]. The effects of butorphanol could be reversed by naloxone (pure opioid antagonist), which acts primarily as a μ-opioid antagonist, but antagonist effects can also occur at κ- and δ-opioid receptors [19]. Furthermore, atipamezole may be used as reversal agent for analgesic procedures when butorphanol or κ-agonists are administered, thereby reducing the recovery time and side effects of butorphanol [20]. Previous studies in calves [21] and horses [22] revealed that epidural butorphanol in combination with lidocaine furnished a long duration of analgesia; however, until now there is dearth of information on the efficacy and safety of epidural butrophanol in camels. Accordingly, the aims of the present study were to comparatively evaluate the onset and duration of analgesia produced by the epidural administration of butorphanol, lidocaine, and butorphanol-lidocaine combination in dromedary camels and monitor their effects on the hemato-biochemical profiles and the time course of heart rate, respiratory rate, and body temperature.

## Results

In all camels, the site of epidural injection was effortlessly identified and the needle was advanced towards the epidural space successfully without complications. Negative aspiration was attempted before injection to confirm accuracy and avoid piercing of the venous sinus.

Complete loss of sensation (score = 3) was achieved in all camels received lidocaine, butorphanol or butorphanol-lidocaine mixture; however, the onset and duration of analgesia differed significantly among treatments (Table 1). The onset of analgesia was earlier and almost similar in animals received lidocaine (6.5 ± 2.3 min) and butorphanol-lidocaine mixture (7.3 ± 1.5 min), while animals given butorphanol showed a significant (*p *< 0.001) delayed onset of analgesia (14.7 ± 3.5 min). Concerning duration, butorphanol treated animals showed a significant (*P* < 0.001) longer period (158 ± 5.3 min) of analgesia than did lidocaine (55 ± 6.8 min) and the butorphanol-lidocaine mixture revealed the longest duration (175 ± 8.7 min) among treatments (*P* < 0.001). Pronounced analgesia was detected in the perineum, base of tail and the caudal upper aspects of the hind limbs in all treatments. However, in butorphanol and butorphanol-lidocaine treated animals, the loss of sensation extended to involve the inguinal region.

**Table 1:** Median (range) of analgesia scores pre- and post-epidural administration of lidocaine hydrochloride 2% (0.22 mg kg^−1^), butorphanol tartarate 1% (0.04 mg kg^−1^) and butorphanol- lidocaine (0.04 mg kg^−1^—0.22 mg kg^−1^) in nine dromedary camels

	Baseline	5	10	15	30	45	60	75	90	105	120	135	150	165	180
LD	0 (0)	1 (0–1)^a^	2 (2–3)^b^	3 (3)^d^	3 (3)^d^	3 (3)^d^	1 (1–2)^c^	0 (0)	0 (0)	0 (0)	0 (0)	0 (0)	0 (0)	0 (0)	0 (0)
BL	0 (0)	1 (0–1)^a^	2 (1–2)^c^	2 (2–3)^b^	3 (3)^d^	3 (3)^d^	3 (3)^d^	3 (3)^d^	3 (3)^d^	3 (3)^d^	3 (3)^d^	3 (3)^d^	3 (2–3)	2 (1–2)^c^	1(0–1)^a^
BT	0 (0)	0 (0)	0 (0–1)	2 (2–3)^b^	3 (3)^d^	3 (3)^d^	3 (3)^d^	3 (3)^d^	3 (3)^d^	3 (3)^d^	3 (3)^d^	3 (3)^d^	3 (3)^d^	3 (2–3)^e^	2 (1–2)^c^

*LD* lidocaine hydrochloride 2%, *BT* butorphanol tartarate 1%, *BL* butorphanol- lidocaine combination (0.04 mg kg-1—0.22 mg kg-1)

^a^^−^^e^ Variables with different superscript letters at the same column are significantly differ at *P* < 0.05

Slight hind limb instability (score = 1) was observed in camels received epidural lidocaine and butorphanol-lidocaine combination (Table 2). Camels treated with lidocaine showed significant shorter duration of ataxia (38 ± 5 min) than those received the butorphanol-lidocaine combination (135 ± 7 min). Camels received epidural butorphanol had normal gait throughout the study (score = 0). All camels given epidural butorphanol and butorphanol-lidocaine combination were slightly sedated (score = 1), while animals received lidocaine showed no signs of sedation (score = 0; Table 3).

**Table 2:** Median (range) of ataxia scores pre- and post-epidural administration of lidocaine hydrochloride 2% (0.22 mg kg-1) butorphanol tartarate 1% (0.04 mg kg-1) and butorphanol- lidocaine (0.04 mg kg-1–0.22 mg kg-1) in nine dromedary camels

	Baseline	5	10	15	30	45	60	75	90	105	120	135	150	165	180
LD	0 (0)	0 (0)	0 (0–1)^a^	1 (0–1)^b^	1 (1)^c^	1 (1)^c^	1 (0–1)^b^	0 (0–1)^a^	0 (0)	0 (0)	0 (0)	0 (0)	0 (0)	0 (0)	0 (0)
BL	0 (0)	0 (0)	0 (0–1)^a^	0 (0–1)^a^	1 (0–1)^b^	1 (1)^c^	1 (1)^c^	1 (1)^c^	1 (1)^c^	1 (1)^c^	1 (1)^c^	1 (1)^c^	1 (0–1)^b^	0 (0–1)^a^	0 (0)
BT	0 (0)	0 (0)	0 (0)	0 (0)	0 (0)	0 (0)	0 (0)	0 (0)	0 (0)	0 (0)	0 (0)	0 (0)	0 (0)	0 (0)	0 (0)

*LD* lidocaine hydrochloride 2%, *BT* butorphanol tartarate 1%, *BL* butorphanol- lidocaine combination (0.04 mg kg-1—0.22 mg kg-1)

^a^^−^^c^ Variables with different superscript letters at the same column are significantly differ at *P* < 0.05

**Table 3:** Median (range) of sedation scores pre- and post-epidural administration of lidocaine hydrochloride 2% (0.22 mg kg^−1^), butorphanol tartarate 1% (0.04 mg kg^−1^) and butorphanol- lidocaine (0.04 mg kg^−1^–0.22 mg kg^−1^) in nine dromedary camels

	Baseline	5	10	15	30	45	60	75	90	105	120	135	150	165	180
LD	0 (0)	0 (0)	0 (0)	0 (0)	0 (0)	0 (0)	0 (0)	0 (0)	0 (0)	0 (0)	0 (0)	0 (0)	0 (0)	0 (0)	0 (0)
BT	0 (0)	0 (0)	0 (0)	1 (0–1)^a^	1 (0–1)^a^	1 (1)^b^	1 (1)^b^	1 (1)^b^	1 (1)^b^	1 (1)^b^	1 (1)^b^	1 (1)^b^	1 (0–1)^a^	1 (0–1)^a^	0 (0–1)^c^
BL	0 (0)	0 (0)	0 (0)	1 (0–1)^a^	1 (0–1)^a^	1 (1)^b^	1 (1)^b^	1 (1)^b^	1 (1)^b^	1 (1)^b^	1 (1)^b^	1 (1)^b^	1 (0–1)^a^	1 (0–1)^a^	1 (0–1)^a^

*LD* lidocaine hydrochloride 2%, *BT* butorphanol tartarate 1%, *BL* butorphanol- lidocaine combination (0.04 mg kg-1—0.22 mg kg-1)

^a^^−^^c^ Variables with different superscript letters at the same column are significantly differ at *P* < 0.05

In all camels, signs of discomfort, ruminal tympany or impaction were not observed during the study. Passing of the first fecal piles was observed approximately one hour post-epidural injection, regardless the injected agent.

There were no significant (*P* > 0.05) changes in the heart rate, respiratory rate and rectal temperature values from the baseline values, between and within treatments. The hematological parameters including the red blood cell (RBCs) count, the hemoglobin concentration (Hb), the packed cell volume (PCV) and the total white blood cell (WBCs) count,were not significantly different (*P* > 0.05) from the baseline values in all treatments.

A transient significant increase in the glucose levels was observed after all treatments. The rise in glucose level was observed 30 min post-epidural administration of lidocaine (from 75.80 ± 8.65 to 97.52 ± 6.23) and 60 min after butorphanol (from 80.52 ± 6.89 to 102.80 ± 7.15) and butorphanol-lidocaine (from 65.39 ± 8.32 to 92.17 ± 8.56) treatments. Other biochemical variables including alanine aminotransferase (ALT), aspartate aminotransferase (AST), blood urea nitrogen (BUN), creatinine (Cr) and electrolytes (Sodium, Na + ; Potassium, K + ; and calcium, Ca + +), were not significantly altered (*p* < 0.05) compared with the baseline values in all treatments.

## Discussion

As far as the authors aware, this is the first study reporting the use of butorphanol as an epidural analgesic agent in dromedary camels. Results of the present study revealed that the period of epidural analgesia was greatly extended by administering butorphanol (synthetic opioid) together with lidocaine (local anesthetic) in dromedary camels. Similar findings were reported in horses, where the period of epidural analgesia was extended by seven folds when butorphanol and lidocaine were given together (136 min), compared to lidocaine alone (36 min) [22]. It seemed that addition of an opioid would account for intensifying and prolonging the loco-regional blockade provided by the local anesthetic agents [23, 24].

In this study, epidural butorphanol showed delayed onset of analgesia compared with butorphanol—lidocaine mixture or lidocaine and the butorphanol—lidocaine mixture had the longest duration of analgesia. This might be attributed to the poor lipid solubility and sluggish dispersion of butorphanol towards the opioid receptors in the dorsal horn of the spinal cord causing slower onset, slower clearance and subsequently long-lasting analgesia [25, 26]. The potentiated analgesic effects of butorphanol—lidocaine mixture may also due to synergy and multimodal analgesic mechanisms of lidocaine and butorphanol [26].

An appropriate epidural procedure should be harmless and devoid of locomotor disturbances or recumbency. As epidural opioids induce analgesia without blocking of the spinal motor functions, it is judicious to assume that no adverse effects on gait would be associated with the pharmacological actions of butorphanol [6]. Results of the present study accentuate the epidural co-administration of butorphanol as a safe analgesic agent, where camels received butorphanol did not develop ataxia or any other adverse effects after the epidural treatment. The signs of mild motor disturbances reported in camels received lidocaine and butorphanol – lidocaine treatments could be attributed to the sensory and motor blockade induced by lidocaine [22]. In calves, the subarachnoid administration of butorphanol – lidocaine mixture induced serious motor disturbances and animals were reluctant to stand during the study [21]. This could be related to the use of a higher dose of lidocaine (4 mg kg^−1^), the different route of administration (subarachnoid route) or the species difference. In sheep, the intra-thecal administration of butorphanol was accompanied by adverse behavioral changes and signs of neurological disorders [27].

In the current study, camels given epidural butorphanol and butorphanol – lidocaine mixture exhibited signs of slight sedation in the form of mild change in the head position and mild drooping of the lower lip. Those signs could be due to the forward dispersal of butorphanol into the central circulation or cranial dissemination of butorphanol into the central nervous system via the cerebrospinal fluid resulting into systemic effects (sedation). Our findings are in congruent with previous studies in horses [22] and calves [21].

The frequency of respiration, the number of heart beats, and the rectal temperature measurements in this study were not significantly altered in comparison with the baseline values in all camels throughout the study. This is largely matching with other studies, where no cardiovascular alterations were observed after epidural administration of butorphanol in dogs [28] or horses [22]. On the contrary, in calves [21], significant rise in heart rate was reported. The discrepancy in findings between studies may be attributed to the species difference, the dose of the given agent or the route of drug administration.

No significant variations in the hematological or biochemical parameters were reported in all camels post epidural administration of all treatments, compared with the baseline values. These findings are similar to previous outcomes recorded when butorphanol was administrated via the intravenous route in camels [17]. A transient rise in the glucose level was observed after all treatments, which returned to basal levels 24 h later. The increased glucose values may be due to the release of adrenocortical hormones and initiation of glycogenolysis in liver and muscles [21, 29].

We did not observe any signs of tympany or abdominal discomfort due to impaction in any of the camels, and camels passed fecal piles without difficulty post epidural administration of all agents. This contradicts previous studies, where in ponies, butorphanol caused decreased jejunal propulsive motility [30] and in horses, butorphanol resulted into marked decrease in the fecal production accompanied by abdominal discomfort [31]. The inconsistency in findings between studies may be attributable to the physiological difference between ponies, horses and camels, the dose of the injectate or the route of drug administration.

## Conclusions

According to our findings, butorphanol produced analgesia of longer duration and delayed onset. Co-administration of butorphanol and lidocaine resulted into rapid onset and considerable long-lasting analgesia extended to the inguinal region. Butorphanol and butorphanol-lidocaine combination revealed satisfactory sedation and ataxia scores. The butorphanol-lidocaine combination is regarded as safe in dromedaries because it did not cause any cardiopulmonary adverse effects, neurological symptoms, ruminal tympany or impaction. A transient increase in glucose level was observed after all treatments that improved during the study as the effects of the drugs wore off. Comprehensive obstetrical manipulations and surgical procedures involving the perineum and inguinal area would be made possible by the prolonged period of analgesia offered by the butorphanol-lidocaine combination, which would also help to alleviate postoperative discomfort.

## Methods

### Animals

Nine healthy mature dromedary camels (5 males and 4 non-pregnant females) were enrolled in this study. Camels were belonging to the Camel Research Center of King Faisal University, KSA. The mean ± SD body weight was 550 ± 56 kg, and age was 9.5 ± 2.5 years. Selected camels were manageable and easy to work with without sedation; free from physical abnormalities; without clinical lameness; and had normal biochemical and hematological profiles. Camels were housed in a corral with free access to food and water. Food was withheld 24 h prior to the experiment.

### Study design

Three different treatments were given to each camel, with a minimum 14-days interval between each treatment (crossover study design). Treatments were: lidocaine hydrochloride 2% (0.22 mg kg^−1^; 1.1 mL 100 kg^−1^; B. Braun Melsungen AG, Germany); butorphanol tartarate 1% (0.04 mg kg^−1^; 0.4 mL 100 kg^−1^; Torbugesic® Zoetis GmbH, Germany) and butorphanol tartarate 1% (0.04 mg kg^−1^) plus lidocaine hydrochloride 2% (0.22 mg kg^−1^). The ultimate volume of each treatment was homogenized to be 10 mL adding sterile water. Treatments were administered gently in order to standardize the pharmaceutical dispersal within the epidural space. Each treatment was given by the same investigator who was unaware to the nature of the given agent.

### Caudal epidural procedure

Camels were placed in sternal recumbency and limbs were secured. The skin over the sacro-coccygeal region was cleansed and aseptically prepared. The epidural space, between the first and second coccygeal vertebrae, was recognized by detecting the first moveable point caudal to the sacrum while moving the tail in ventro-dorsal directions. The epidural space was reached by inserting an 18-gauge standard needle in a ventro-cranial direction at an angle of 45° relative to the horizontal plane until crossing the ligamentum flavum. Before injection into the epidural space, aspiration was attempted to ensure that a venous sinus was not accidentally pierced. After injection, camels were led to a chute and observed.

### Assessment

During the experiment, clinical data were collected by a researcher who was unaware to the name of the drug being tested. Each individual assessment was conducted by the same investigator in each treatment. Animals were blindfolded during assessment to avoid their training to stimulations. The degree and intensity of analgesia and the length of time it lasted, as well as the motor effects and degree of sedation, were evaluated and reported. Baseline (0 min) clinical and hemato-biochemical values were recorded for each animal before each treatment and were set as control values.

The onset time of analgesia was designed as the time elapsed from the epidural administration of the injectate until the beginning of numbness. The duration was set as the period between the start of insensibility to the return of the usual response in the area of interest. The presence of pain was tested by 21-gauge needle and by closing of a hemostatic forceps to the first ratchet in the tail, perineum, genital organs, inguinal region, and thighs and continued towards the umblical region. Responses were tested every single minute until complete loss of sensation, and then repeated at 5 min interval.

The grade of insensibility (analgesia) was classified using a 0‒3 scale [32]: 0 = usual or full response; 1 = weak reaction to the applied stimulus; 2 = moderate analgesia or slight avoidance reaction; and 3 = complete insensibility.

The degree of locomotor disturbance (ataxia) was evaluated every 15-min intervals during the experiment by observing the gait of camels when they were led out of the stanchions. Ataxia was graded on a 0‒3 scale [32] as follows: 0 = normal gait; 1 = slight stumbling but can walk easily; 2 = imbalance with very ataxic gait; and 3 = completely recumbent.

Sedation was also evaluated every 15-min intervals as for ataxia and was graded on a 0–3 scale [32]: 0 = fully responsive to surroundings with normal position of lips, head and ears; 1 = mildly sedated, still responsive with slight head bowing and minor separation of the lower lip; 2 = moderately sedated, unaware of surroundings with muzzle drooping and lowered head and ears; 3 = deeply sedated, unaware of the surroundings with marked head drooping below the level of the shoulders and drooping of lips and eyelids.

Heart rate was assessed by counting heart beats per minute over the cardiac area using a stethoscope. The frequency of respiration was estimated by counting chest movements per minute and the rectal temperature was measured by a digital thermometer (^∘^C). The heart rate, respiratory rate, and rectal temperature were recorded before (baseline, 0) and at 15-min intervals in each treatment. The incidence of defecation and the time to passage of the first pile of manure after epidural injection were recorded.

### Blood sampling

The skin covering the jugular groove was aseptically prepared and an intravenous catheter was inserted into the jugular vein and secured. Samples were collected prior to (baseline, 0-min) and at 15, 30, 60, 120, 180 min and then 24 h after each treatment. Blood samples were subjected to hematological (total RBCs count, Hb concentration, PCV and the total WBCs count) and biochemical (glucose level, ALT, AST, BUN, Cr, Na + , K + and Ca + +) analyses.

### Statistical analysis

Statistical analyses were accomplished using a statistical SPSS for Windows version 22 (SPSS Ltd., IL, USA). Parametric variables were analyzed using the general linear models procedure for analysis of variance. One-way ANOVA accompanied with Duncan’s multiple range tests were used to detect the differences among means; data are shown as mean ± SD. The nonparametric one-way test was applied to detect the difference in scores throughout the time points. Therefore, Friedman’s chi squared estimated by Cochran–Mantel–Haenszel analysis, within repeated measurements was applied. The signed rank test (Wilcoxon two-sample test for paired observations) within univariate analysis was used for post hoc estimation of pair wise differences between two time points. Values of *p* < 0.05 were considered significant. Data are presented as median (range).

## Supplementary Information

**Additional file 1: Supplementary Table 1. **Mean values ± SD of clinical parameters (heart rate, respiratory rate and rectal temperature) pre- and post-epidural administration of lidocaine hydrochloride 2% (0.22 mg kg^-1^); butorphanol tartarate 1% (0.04 mg kg^-1^) and butorphanol- lidocaine (0.04 mg kg^-1^-0.22 mg kg^-1^)in nine dromedary camels.

**Additional file 2: Supplementary Table 2. **Mean values ± SD of hematological variables pre- and post-epidural administration of lidocaine hydrochloride 2% (0.22 mg kg-1), butorphanol tartarate 1% (0.04 mg kg-1) and butorphanol- lidocaine (0.04 mg kg-1-0.22 mg kg-1) in nine dromedary camels.

**Additional file 3: Supplementary Table 3. **Mean values ± SD of biochemical variables pre- and post-epidural administration of lidocaine hydrochloride 2% (0.22 mg kg-1), butorphanol tartarate 1% (0.04 mg kg-1) and butorphanol- lidocaine (0.04 mg kg-1-0.22 mg kg-1) in nine dromedary camels.

## Data Availability

The datasets used and/or analyzed during the current study are available from the corresponding author on reasonable request.

## Abbreviations

RBCs: Red blood cells
WBCs: White blood cells
Hb: Hemoglobin concentration
PCV: Packed cell volume
ALT: Alanine aminotransferase enzyme
AST: Aspartate aminotransferase enzyme
BUN: Blood urea nitrogen
Cr: Creatinine
Na +: Sodium
K +: Potassium and
Ca + +: Calcium
