# Supplementary material for: Epidural lidocaine, butorphanol, and butorphanol – lidocaine combination in dromedary camels
Source: BMC Vet Res. 2023 Feb 16;19:51. doi: 10.1186/s12917-023-03601-8 (PMC9933277; doi:10.1186/s12917-023-03601-8)
Supplement: Supplementary file 1 — Additional file 1: Supplementary Table 1. Mean values ± SD of clinical parameters (heart rate, respiratory rate and rectal temperature) pre- and post-epidural administration of lidocaine hydrochloride 2% (0.22 mg kg-1); butorphanol tartarate 1% (0.04 mg kg-1) and butorphanol- lidocaine (0.04 mg kg-1-0.22 mg kg-1)in nine dromedary camels. [file 12917_2023_3601_MOESM1_ESM.docx]

**Supplementary Table 1**: Mean values ± SD of clinical parameters (heart rate, respiratory rate and rectal temperature) pre- and post-epidural administration of lidocaine hydrochloride 2% (0.22 mg kg^-1^); butorphanol tartarate 1% (0.04 mg kg^-1^) and butorphanol- lidocaine (0.04 mg kg^-1^-0.22 mg kg^-1^) in nine dromedary camels.

| Time  (Min) | Heart Rate (beat/min) | | | Respiratory Rate (breath/min) | | | Rectal Temperature (°C) | | |
| --- | --- | --- | --- | --- | --- | --- | --- | --- | --- |
|  | LD | BT | BL | LD | BT | BL | LD | BT | BL |
| Baseline | 46 ± 5 | 43 ± 5 | 46 ± 3 | 10 ± 4 | 11 ± 2 | 9 ± 4 | 36.3 ± 0.8 | 36.8 ± 0.5 | 37 ± 0.2 |
| 15 | 46 ± 3 | 44 ± 2 | 47 ± 5 | 10 ± 3 | 12 ± 3 | 10 ± 2 | 36.2 ± 0.5 | 36.8 ± 0.5 | 36.9 ± 0.1 |
| 30 | 46 ± 2 | 44 ± 3 | 47 ± 5 | 11 ± 3 | 12 ± 1 | 10 ± 2 | 36.2 ± 0.4 | 36.3 ± 0.2 | 36.9 ± 0.2 |
| 45 | 45 ± 6 | 44 ± 3 | 47 ± 3 | 10 ± 2 | 11 ± 1 | 10 ± 1 | 36.1 ± 0.2 | 36.5 ± 0.5 | 36.9 ± 0.2 |
| 60 | 45 ± 3 | 44 ± 4 | 47 ± 2 | 10 ± 3 | 11 ± 2 | 11 ± 0 | 36.0 ± 0.3 | 36.9 ± 0.2 | 37.0 ± 0.5 |
| 75 | 45 ± 3 | 43 ± 2 | 46 ± 4 | 10 ± 2 | 11 ± 2 | 10 ± 1 | 36.0 ± 0.7 | 36.8 ± 0.1 | 37.1 ± 0.5 |
| 90 | 46 ± 5 | 43 ± 5 | 46 ± 1 | 10 ± 1 | 11 ± 1 | 10 ± 1 | 36.0 ± 0.7 | 36.0 ± 0.5 | 37.2 ± 0.2 |
| 105 | 45 ± 2 | 43 ± 5 | 46 ± 4 | 10 ± 2 | 11 ± 1 | 9 ± 2 | 36.5 ± 0.4 | 36.0 ± 0.1 | 37.0 ± 0.4 |
| 120 | 45 ± 3 | 44 ± 2 | 46 ± 4 | 10 ± 3 | 12 ± 2 | 10 ± 0 | 36.5 ± 0.5 | 36.5 ± 0.5 | 36.8 ± 0.4 |
| 135 | 46 ± 3 | 44 ± 2 | 45 ± 3 | 10 ± 3 | 12 ± 1 | 11 ± 0 | 36.4 ± 0.2 | 36.3 ± 0.1 | 36.9 ± 0.5 |
| 150 | 45 ± 5 | 43 ± 4 | 46 ± 6 | 11 ± 4 | 11 ± 0 | 11 ± 1 | 36.2 ± 0.5 | 36.2 ± 0.2 | 37.0 ± 0.5 |
| 165 | 45 ± 4 | 43 ± 4 | 46 ± 4 | 10 ± 3 | 11 ± 1 | 9 ± 2 | 36.0 ± 0.6 | 36.6 ± 0.7 | 37.0 ± 0.0 |
| 180 | 45 ± 4 | 43 ± 3 | 47 ± 4 | 10 ± 4 | 11 ± 3 | 9 ± 3 | 36.3 ± 0.1 | 36.6 ± 0.6 | 37 ± 0.0 |

LD, lidocaine HCL 2%; BT, butorphanol tartarate 1%; BL, butorphanol - lidocaine combination. Variables at the same
 column are significantly different at P < 0.05.
