# Supplementary material for: Epidural lidocaine, butorphanol, and butorphanol – lidocaine combination in dromedary camels
Source: BMC Vet Res. 2023 Feb 16;19:51. doi: 10.1186/s12917-023-03601-8 (PMC9933277; doi:10.1186/s12917-023-03601-8)
Supplement: Supplementary file 2 — Additional file 2: Supplementary Table 2. Mean values ± SD of hematological variables pre- and post-epidural administration of lidocaine hydrochloride 2% (0.22 mg kg-1), butorphanol tartarate 1% (0.04 mg kg-1) and butorphanol- lidocaine (0.04 mg kg-1-0.22 mg kg-1) in nine dromedary camels. [file 12917_2023_3601_MOESM2_ESM.docx]

**Supplementary Table 2**: Mean values ± SD of hematological variables pre- and post-epidural administration of lidocaine hydrochloride 2% (0.22 mg kg-1), butorphanol tartarate 1% (0.04 mg kg-1) and butorphanol- lidocaine (0.04 mg kg-1-0.22 mg kg-1) in nine dromedary camels.

| Variables | Treatments | Baseline | Time/minute | | | | | 24 hours |
| --- | --- | --- | --- | --- | --- | --- | --- | --- |
|  |  |  | 15 | 30 | 60 | 120 | 180 |  |
| RBCs x 10^6^/mm^3^ | LD | 7.83 ± 2.82 | 7.86 ± 2.66 | 7.71 ± 2.93 | 7.77 ± 2.63 | 7.5 ± 3.1 | 7.88 ± 2.55 | 7.65 ± 2.89 |
|  | BT | 9.55 ± 3.44 | 9.30 ± 3.12 | 9.35 ± 3.40 | 9.20 ± 3.23 | 9.20 ± 3.50 | 9.45 ± 3.42 | 9.45 ± 3.65 |
|  | BL | 8.42 ± 2.25 | 8.20 ± 3.22 | 8.40 ± 2.40 | 8.35 ± 1.95 | 8.49 ± 2.55 | 8.43 ± 2.36 | 8.50 ± 2.66 |
| WBCs x 10^6^/mm^3^ | LD | 11.33 ± 6.23 | 11.00 ± 5.22 | 11.20 ± 5.23 | 11.30 ± 6.10 | 11.10 ± 5.90 | 11.40 ± 6.00 | 11.37 ± 4.69 |
|  | BT | 10.66 ± 4.22 | 10.22 ± 3.59 | 10.34 ± 4.00 | 10.52 ± 4.40 | 10.70 ± 4.30 | 10.60 ± 4.60 | 10.85 ± 4.79 |
|  | BL | 11.82 ± 2.45 | 11.72 ± 2.29 | 11.63 ± 3.32 | 11.72 ± 2.54 | 11.74 ± 2.23 | 11.80 ± 2.35 | 11.77 ± 3.25 |
| Hb g/dl | LD | 9.85 ± 0.42 | 9.23± 0.88 | 9.20 ± 0.55 | 9.70 ± 0.45 | 9.70 ± 0.26 | 9.77 ± 0.82 | 9.82 ± 0.44 |
|  | BT | 10.36 ± 1.50 | 10.44 ± 0.98 | 10.45 ± 1.23 | 10.32 ± 1.37 | 10.39 ± 1.69 | 10.45 ± 1.88 | 10.40 ± 1.60 |
|  | BL | 12.45 ± 0.88 | 12.5 ± 0.80 | 11.95 ± 0.85 | 12.25 ± 0.90 | 12.25 ± 0.75 | 12.42 ± 0.70 | 12.50 ± 0.65 |
| PCV % | LD | 26.77 ± 1.62 | 26.60 ± 1.50 | 26.12 ± 1.89 | 26.75 ± 1.45 | 26.68 ± 1.20 | 26.70 ± 1.52 | 26.78 ± 1.60 |
|  | BT | 28.32 ± 1.79 | 28.32 ± 1.79 | 28.40 ± 1.69 | 28.30 ± 1.72 | 28.20 ± 1.82 | 28.45 ± 1.65 | 28.36 ± 1.75 |
|  | BL | 25.32 ± 0.77 | 25.35 ± 0.44 | 25.42 ± 0.70 | 25.21 ± 0.80 | 25.43 ± 0.80 | 25.56 ± 0.82 | 25.39 ± 0.76 |

LD, lidocaine HCL 2%; BT, butorphanol tartarate 1%; BL, butorphanol - lidocaine combination; RBCs, red blood cells; WBCs, white blood cells; Hb, hemoglobin; PCV, packed cell volume. Significantly different parameter from baseline at each group (p < 0.05).
