# Supplementary material for: Epidural lidocaine, butorphanol, and butorphanol – lidocaine combination in dromedary camels
Source: BMC Vet Res. 2023 Feb 16;19:51. doi: 10.1186/s12917-023-03601-8 (PMC9933277; doi:10.1186/s12917-023-03601-8)
Supplement: Supplementary file 3 — Additional file 3: Supplementary Table 3. Mean values ± SD of biochemical variables pre- and post-epidural administration of lidocaine hydrochloride 2% (0.22 mg kg-1), butorphanol tartarate 1% (0.04 mg kg-1) and butorphanol- lidocaine (0.04 mg kg-1-0.22 mg kg-1) in nine dromedary camels. [file 12917_2023_3601_MOESM3_ESM.docx]

**Supplementary Table 3**: Mean values ± SD of biochemical variables pre- and post-epidural administration of lidocaine hydrochloride 2% (0.22 mg kg-1), butorphanol tartarate 1% (0.04 mg kg-1) and butorphanol- lidocaine (0.04 mg kg-1-0.22 mg kg-1) in nine dromedary camels.

| Variables | Treatments | Baseline | Time/minute | | | | | 24 hours |
| --- | --- | --- | --- | --- | --- | --- | --- | --- |
|  |  |  | 15 | 30 | 60 | 120 | 180 |  |
| Glucose  (mg dL^−1^) | LD | 75.80 ± 8.65 | 75.75 ± 7.26 | **97.52 ± 6.23** | 75.78 ± 7.55 | 75.60 ± 6.26 | 75.20± 5.89 | 75.70 ± 7.24 |
|  | BT | 80.52 ± 6.89 | 80.89 ± 8.50 | 80.85 ± 5.56 | **102.80 ± 7.15** | 80.89 ± 6.90 | 81.11 ± 5.24 | 80.88 ± 7.12 |
|  | BL | 65.39 ± 8.32 | 66.79 ± 9.15 | 65.85 ± 8.72 | **92.17 ± 8.56** | 65.56 ± 7.72 | 66.42 ± 7.23 | 65.18 ± 9.15 |
| BUN  (mg dL^−1^) | LD | 12.54 ± 1.56 | 13.22 ± 1.80 | 13.25 ± 1.75 | 12.89 ± 1.25 | 12.85 ± 1.89 | 12.75 ± 1.62 | 12.65 ± 1.52 |
|  | BT | 10.89 ± 2.42 | 10.95 ± 3.55 | 10.98 ± 2.85 | 10.45 ± 2.56 | 10.82 ± 2.52 | 10.92 ± 2.52 | 10.81 ± 2.78 |
|  | BL | 15.47 ± 2.30 | 15.78 ± 3.35 | 15.85 ± 3.12 | 15.35 ± 2.89 | 15.58 ± 2.74 | 15.23 ± 2.18 | 15.54 ± 2.65 |
| Cr  (mg dL^−1^) | LD | 0.88 ± 0.12 | 0.89 ± 0.15 | 0.89 ± 0.18 | 0.88 ± 0.14 | 0.88 ± 0.16 | 0.89 ± 0.13 | 0.89 ± 0.15 |
|  | BT | 1.12 ± 0.31 | 1.17 ± 0.42 | 1.16 ± 0.35 | 1.18 ± 0.39 | 1.22 ± 0.41 | 1.23 ± 0.21 | 1.25 ± 0.31 |
|  | BL | 0.98 ± 0.44 | 0.98 ± 0.35 | 0.99 ± 0.56 | 0.99 ± 0.52 | 0.99 ± 0.42 | 0.98 ± 0.35 | 0.98 ± 0.52 |
| ALT  (IU L^−1^) | LD | 18.91 ± 3.52 | 18.52 ± 3.23 | 18.55 ± 5.25 | 18.80 ± 4.61 | 18.42 ± 3.52 | 18.51 ± 6.31 | 18.20 ± 6.92 |
|  | BT | 10.52 ± 2.53 | 10.54 ± 3.52 | 10.10 ± 3.89 | 10.85 ± 4.45 | 10.92 ± 8.93 | 11.95 ± 6.54 | 10.98 ± 5.23 |
|  | BL | 12.92 ± 3.55 | 12.90 ± 3.51 | 12.95 ± 2.24 | 12.98 ± 3.52 | 12.56 ± 7.67 | 12.70 ± 5.82 | 12.71 ± 4.84 |
| AST  (IU L^−1^) | LD | 37.54 ± 8.84 | 37.22 ± 6.61 | 37.43 ± 5.50 | 37.82 ± 9.54 | 37.21 ± 8.54 | 37.62 ± 5.94 | 37.82 ± 5.66 |
|  | BT | 45.23 ± 5.81 | 45.2 ± 8.25 | 45.30 ± 6.3 | 45.42 ± 2.26 | 45.55 ± 7.47 | 45.67 ± 7.55 | 44.82 ± 9.53 |
|  | BL | 42.56 ± 8.80 | 42.54 ± 8.85 | 42.52 ± 4.53 | 42.52 ± 6.25 | 42.39 ± 6.57 | 42.67 ± 6.60 | 42.85 ± 9.50 |
| Ca^2+^  (mg dL^−1^) | LD | 9.87 ± 1.47 | 9.52 ± 1.56 | 9.99 ± 1.32 | 9.85 ± 1.45 | 9.80± 1.35 | 9.85 ± 1.56 | 9.90 ± 1.45 |
|  | BT | 8.96 ± 1.36 | 8.92 ± 1.52 | 8.90 ± 1.30 | 8.88 ± 1.48 | 8.75 ± 1.45 | 8.91 ± 1.41 | 8.87 ± 1.43 |
|  | BL | 8.45 ± 1.88 | 8.59 ± 1.85 | 8.74 ± 1.62 | 8.84 ± 1.80 | 8.92 ± 1.74 | 8.88 ± 1.56 | 8.59 ± 1.69 |
| Na^+^  (mmol L^−1^) | LD | 155.65 ± 12.50 | 156.25 ± 15.12 | 155.25 ± 12.13 | 155.85 ± 11.40 | 155.00 ± 13.40 | 156.70 ± 11.90 | 155.90 ± 12.84 |
|  | BT | 145.84 ± 18.42 | 144.99 ± 17.12 | 145.75 ± 15.72 | 145.94 ± 19.52 | 145.90 ± 19.61 | 145.92 ± 16.60 | 145.94 ± 17.49 |
|  | BL | 162.30 ± 15.23 | 162.70 ± 14.33 | 163.40 ± 16.26 | 163.20 ± 17.11 | 163.20 ± 16.69 | 162.88 ± 14.49 | 162.67 ± 17.52 |
| K^+^  (mmol L^−1^) | LD | 3.62 ± 0.45 | 3.75 ± 0.50 | 3.72 ± 0.62 | 3.69 ± 0.43 | 3.60 ± 0.40 | 3.68 ± 0.62 | 3.65 ± 0.35 |
|  | BT | 4.25 ± 0.88 | 4.49 ± 0.75 | 4.85 ± 0.91 | 4.57 ± 0.62 | 4.69 ± 0.96 | 4.72 ± 0.57 | 4.88 ± 0.61 |
|  | BL | 4.45 ± 0.96 | 4.69 ± 0.78 | 4.72 ± 0.88 | 4.56 ± 0.57 | 4.70 ± 0.92 | 4.55 ± 0.93 | 4.63 ± 0.94 |

LD, lidocaine HCL 2%; BT, butorphanol tartarate 1%; BL, butorphanol - lidocaine combination; Na+ , sodium; K+ , potassium; Ca2+ , calcium; Cr, creatinine; BUN, blood urea nitrogen; ALT, alanine transaminase; AST, aspartate transaminase. Bold mean values indicate significant differences (p < 0.05) in mean values within groups compared to baseline values.
